# Dietary artemisinin boosts intestinal immunity and healthy in fat greenling (*Hexagrammos otakii*)

**DOI:** 10.3389/fimmu.2023.1198902

**Published:** 2023-07-17

**Authors:** Yixin Gu, Wenjie Wang, Yu Zhan, Xiaoyan Wei, Yanyan Shi, Dandan Cui, Tingting Peng, Jian Han, Xuejie Li, Yan Chen, Zhuang Xue, Wei Wang

**Affiliations:** Key Laboratory of Applied Biology and Aquaculture of Northern Fishes in Liaoning Province, Dalian Ocean University, Dalian, China

**Keywords:** artemisinin, intestinal immune disease, network pharmacology, molecular docking, *Hexagrammos otakii*

## Abstract

**Introduction:**

Artemisinin (ART) is very common as a diet additive due to its immunoregulatory activities. Nonetheless, the immunoregulatory mechanism of ART in marine fish remains unknown. This study comprehensively examined the effects and explored the potential mechanism of ART ameliorating intestinal immune disease (IID) in fat greenlings *(Hexagrammos otakii)*.

**Methods and results:**

The targets of ART were screened using the Traditional Chinese Medicine Systems Pharmacology (TCMSP) database. Here, eight putative targets of ART were collected and identified with the Uniprot database, and 1419 IID-associated target proteins were filtered through the Drugbank, Genecards, OMIM, and PHARMGKB Databases. The results of Gene Ontology (GO) and Kyoto Encyclopedia of Genes and Genomes (KEGG) pathways point out that ART may have immunoprotective effects by regulating cellular responses to stress, hypoxia, inflammation, and vascular endothelial growth factor stimulus through the hypoxia-inducible factor 1 (HIF-1) signaling pathway. The findings of molecular docking indicated that ART contains one active ingredient and three cross-targets, which showed a kind combination with hypoxia-inducible factor 1-alpha (HIF1-a), transcription factor p65 (RELA), and vascular endothelial growth factor A (VEGF-A), respectively. Furthermore, an ART feeding model was established to assess the ART’s immunoprotect effect on the intestine of *H.otakii in vivo*. The D48 group showed smaller intestinal structural changes after being challenged by *Edwardsiella tarda*. The supplementation of ART to the diet improved total superoxide dismutase (SOD), catalase (CAT), and glutathione peroxidase (GSH-Px) and reduced the malondialdehyde (MDA) in intestine of *H. otakii*. The expression of transcription factor p65, HIF1-α, VEGF-A, cyclin D1, matrix metalloprotease 9 (MMP9), monocyte chemoattractant protein-1 (MCP-1), tumor necrosis factor-alpha (TNF-α), and interleukin-6 (IL-6) was decreased after dietary ART in the intestinal of *H. otakii*.

**Discussion:**

The present results demonstrated that dietary ART improved antioxidants and immunity, optimized the intestinal structure, and increased resistance to *E. tarda* through the SOD2/nuclear-factor-kappa- B (NFkB)/HIF1-a/VEGF-A pathway in the intestinal tract of *H.otakii*. This study integrated pharmacological analysis and experimental validation and revealed the mechanism of ART on IID, which provides insight into the improvement of IID in *H. otakii*.

## 1 Introduction

The fat greenling (*Hexagrammous otakii*) is a species of Scorpaeniformes that is primarily located in China, the Korean Peninsula, and Japan. It is a commercially significant species due to its high nutritional value and superior meat quality (1). In recent years, high-density intensive farming systems have become an increasingly attractive approach to meet the rising demand for this species. Nonetheless, these farming methods subject fish to substantial stress conditions, exacerbating intestinal immune disease (IID), making the fish more prone to disease, and resulting in high mortality rates and significant economic losses (2). Various strategies, including the extensive use of antibiotics such as flavomycin, bacitracin zinc, halomycin, and enomycin, have been employed to treat the disease (3–5). However, antibiotic resistance, environmental pollution, and the accumulation of residues in fish and subsequently human tissues have become substantial obstacles to the growth of intensive aquaculture (6, 7). Accordingly, the quest for environmentally sustainable and safe alternatives to manage fish IID has gradually become the focus of global research endeavors.

Recently, aquatic animal diseases have been controlled by herbal medicine, which is the main direction of modern aquatic animal medicine (8, 9). Artemisinin (ART) is a herbal medicine extracted at low temperatures from *Artemisia annua* (10). It has been shown to be the most effective treatment for malaria while also providing antibacterial (11), immunomodulatory (12), anti-inflammatory (13) properties and has attracted the attention of scholars (14). Currently, the research related to ART for the treatment of IID is concentrated in the medical field. Huai et al. (15) reported that ART can treat IID by modulating macrophage polarization and epithelial interstitial processes. Also, the ART analog SM394 could alleviate dextran sulfate sodium-induced ulcerative colitis by inhibiting neutrophils and macrophages as well as the nuclear factor κB (NFκB) signaling pathway (16). In addition, the supplementation of 2 mg/kg of ART could reduce intestinal inflammation in weaned piglets and enhance intestinal immunity and digestive capacity (17). There has been increasing interest in recent years in improving animal health through the use of ART additives, which have positive effects on various fish species, including common carp (*Cyprinus carpio*) (18), rainbow trout (*Oncorhynchus mykiss*) (19), Nile tilapia (*Oreochromis niloticus*) (20), Mozambique tilapia (*Oreochromis mossambicus*) (Mbokane and Moyo, 2018), African catfish (*Clarias gariepinus*) (21), and largemouth bass (*Micropterus salmoides*) (22). For instance, the supplementation of ART to the diet of *Litopenaeus vannamei* significantly ameliorated *Vibrio parahaemolyticus-*induced intestinal inflammation and poor histomorphology (23). The primary beneficial impacts of ART include the enhancement of fish performance by mitigating the immune system and improving intestinal functionality, nutrient digestibility, and antioxidant capacity (23, 24). However, the pharmacological and molecular mechanisms are poorly understood despite the recognized therapeutic benefits of ART.

Systems pharmacology is a burgeoning field that amalgamates aspects of pharmacology, drug target networks, and pharmacodynamics. The holistic approach it embraces is congruent with the principles of trellis-coded (TCM) modulation (25). However, systems pharmacology has been relatively understudied in the realm of aquaculture. It is noteworthy that our preceding experiments confirmed that dietary ART enhanced the growth performance and nonspecific immunity of *H. otakii* (data not shown). Therefore, this study set out to assess the molecular mechanisms of ART via active compound screening, therapeutic target prediction, and experimental verification to provide the basic data for the research and development of exclusive feed additives for *H. otakii*.

## 2 Materials and methods

### 2.1 Putative target protein and IID- associated protein screening

The artemisinin was searched in the Traditional Chinese Medicine Systems Pharmacology Database (TCMSP, http://tcmspw.com/tcmsp.php) to obtain its corresponding active ingredients and targets, and then these targets were imported into the UniProt database (https://www.uniprot.org/), and the organism was selected for “Zebrafish (*Danio rerio*)”.

The DrugBank database (https://go.drugbank.com), Online Mendelian Inheritance in Man (OMIM) database (https://omim.org), Genecards database (https://www.genecards.org), and Pharmacogenomics Knowledgebase (PHARMGKB) database (https://www.pharmgkb.org) to search for IID-related targets. The active goals were searched from the Genecards database with a relevance score of ≥ 10 as a screening standard.

### 2.2 Gene Ontology and Kyoto Encyclopedia of Genes and Genomes Pathway Enrichment and Network Constructions

The targets were entered into the DAVID database (https://david.ncifcrf.gov) for Gene Ontology (GO) and Kyoto Encyclopedia of Genes and Genomes (KEGG) pathway enrichment analyses. Zebrafish and 0.05 were chosen as the species and *p-*value limit , respectively. The −log10 (*p*)- values for enrichment results were sorted from highest to lowest.

The targets were entered into Cytoscape 3.8.2 software for significance screening. The overlap between ART targets and disease targets was calculated using the Venny platform 2.1.0 (https://bioinfogp.cnb.csic.es/tools/venny), and the intersecting targets were then entered into the STRING 11.0 platform (https://cn.string-db.org) to create a PPI network plot, where the species and “medium confidence” were identified as Zebra fish and 0.400, receptively.

### 2.3 Molecular docking

The three target gene sequences were obtained from the transcriptome database (https://report.majorbio.com/drna/specimen_general/task_id/b5ml_leh1nhp7g5395igcov5puu) of *H. otakii*, and subsequently converted into protein sequences at the National Center for Biotechnology Information (https://www.ncbi.nlm.nih.gov/). The software Alphafold 2 (https://colab.research.google.com/github/sokrypton/ColabFold/blob/main/AlphaFol2.ipynb#) is used to calculate the highly accurate structure through the target protein sequence.

The ART ligand was obtained as a PDB coordinate for *Drosophila* by downloading it from PubChem and using ChemDraw 3D software. The ligand follows Lipinski’s “Rule of Five,” which establishes standards for drug-like characteristics (26). The binding sites were forecast for individual proteins by Deepsite (https://www.playmolecule.com/deepsite/). The binding energy of the ligand–receptor complex was calculated using Auto Dock Vina 1.5.7 software to simulate ligand entry into the active site of the protein. Nine docking positions of each ligand–protein complex were predicted based on computer docking. The protein has a preference for a lower binding affinity score with the preferred binding orientation of this compound, which warrants that it be further investigated. The visualization was performed using Pymol 2.4.0 for three-dimensional (3D) and docking structures and LigPlot 2.2.5 for two-dimensional (2D) structures.

### 2.4 Experimental diets and design

The experiment was conducted by adding 600 mg/kg of ART to the diet in two groups labeled 0 (A0) and 600 mg/kg (D0), respectively, as shown in **Table 1**. All the diet materials were crushed through a 60-mesh sieve, and the ingredients were mixed step by step according to the recipe, during which an appropriate amount of water was added to give them a suitable adhesion and granulated into 2-mm pellets by a granulator. The diet was dried at 43°C to about 10% moisture, sealed in plastic bags, and maintained at −20°C.

**Table 1:** Formulation and proximate composition of the experimental diets (% dry matter).

Ingredients	Dietary ART level (%)	
	A0	D0
Fish meal^a^	40	40
Soybean meal^b^	30	30
Casein^c^	16	16
Fish oil^d^	7	7
Flours^e^	2	2
Corn starch^f^	2	2
Vitamin premix^g^	1	1
Mineral premix^h^	1	1
Sodium alginate^i^	1	1
ART^j^	0	0.06
Total	100	100
Proximate composition (%) moisture	9.54	9.51
Protein	50.23	50.36
Lipid	10.54	10.53
Ash	8.09	7.93

a Crude protein: 58%; crude lipid: 7.2%. Purchased from Meiweiyuan Biotechnology Co., Qingdao, Shandong, China.

b Crude protein: 42.5%, crude lipid: 2.1%. Purchased from Meiweiyuan Biotechnology Co., Qingdao, Shandong, China.

c Crude protein: 86.2%; crude lipid: 1.5%; purchased from Meiweiyuan Biotechnology Co., Qingdao, Shandong, China.

d Fish oil: purchased from Meiweiyuan Biotechnology Co., Qingdao, Shandong, China.

e Crude protein: 6.2%; crude lipid: 0.9%; purchased from Meiweiyuan Biotechnology Co., Qingdao, Shandong, China.

f Crude protein: 0.3%; crude Lipid: 0.1%; purchased from Meiweiyuan Biotechnology Co., Qingdao, Shandong, China.

g Vitamin premix: 7,000 IU of vitamin A; 50 mg of vitamin E; 200 IU of vitamin D_3_; 10 mg of vitamin K_3_; 20 mg of vitamin B_1_; 20mg of vitamin B_2_; 30 mg of vitamin B_6_; 0.1 mg of vitamin B_12_; 80 mg of nicotinic acid; 100 mg of vitamin C; 50 mg of Ca pantothenate; 6 mg of folic acid; 80 mg of inositol (diet per kilogram).

h Mineral premix: 5782 mg of MgSO_4_·7H_2_O; 100 mg of FeSO_4_·7H_2_O; 3,000 mg of NaCl; 150 mg of ZnSO_4_·7H_2_O; 50.3 mg of MnSO_4_·4H_2_O; 15 mg of CuSO_4_·5H_2_O; 1.2 mg of CoCl_2_·6H_2_O; 1.5 mg of KI (diet per kilogram).

i Sodium alginate: purchased from Meiweiyuan Biotechnology Co., Qingdao, Shandong, China.

j Artemisinin (ART): purchased from McLin Biotech Co., Shanghai, China.

### 2.5 Experiment feeding management

*H. otakii* was obtained through artificial breeding at the Key Laboratory of Applied Biology and Aquaculture of Fish (Dalian, China). This experiment followed the regulations of the National Institute for Animal Research and the Animal Experiment Ethics Committee of Dalian Ocean University. A total of 60 healthy juvenile fishes (20.1 ± 0.25 g) were selected and randomly placed into six (30 cm × 75 cm) cages (*n* = 10) in a circulation pond (presterilized). The fish were fasted for 24 h before the experiment, and each diet was fed to three replicate groups. The test fish were temporarily housed for 1 week to adapt to the environmental conditions in the laboratory. For 28 days of the trial, feeding was done (9:00 and 16:00) twice a day and during the natural photoperiod. Parameters include a water temperature of 17.6°C ± 1.5°C, a salinity of 28 –30, a pH of 7.4 ± 0.3, dissolved oxygen of 7.4 ± 0.5 mg/L, and an ammoniacal nitrogen level of no more than 0.1 mg/L. After 4 weeks of rearing, 10 healthy fish per group (10 fish per tank) were treated with 100 mg/L methane-sulfonate-222 (MS-222, Sigma, USA). The intestine was quickly obtained and kept at −80°C.

### 2.6 *Edwardsiella tarda* challenge

The strain of *E. tarda* used in the fish infection experiment was obtained from the Key Laboratory of Seafood Disease Prevention and Control, Dalian Ocean University. The strains were removed from the −80°C state. We used sterile test tubes and added 4 mL of liquid Luria–Bertani (LB) medium and 50 µL of strain. It was then incubated for 12 h in a shaker incubator under sealed conditions at 37°C. The strains were activated, counted according to the procedure, and stored at 4°C until use. Each of the nine sterilized tubes were numbered, and 900 µL of sterile water was added. Test tube 1 should be filled with 100 µL of the activated strain after it has been diluted with sterile water at a ratio of 1:10 and add ed into nine test tubes. The diluted strain in test tubes 7, 8, and 9 should then be removed and spread evenly over the nutrient agar culture dish. The control group was given an equal amount of saline. The strains were then incubated in a constant- temperature incubator for 24 h at 25°C, and the number of colonies and the concentration of the strain suspension were calculated. Finally, the strain suspension at the determined concentration was diluted to the infection concentration. Briefly, each 0.1 mL of strain contains 1 × 10^7^ colony-forming units (CFU) of bacteria. The calculation formula is CFU = the number of colonies × dilution times × 5. After 4 weeks of the feeding trial, the remaining five fish were injected intraperitoneally with *E. tarda* (0.1 mL) and continued to be infected for 48 h (A48 and D48). The fish were then anesthetized with 100 mg/L MS-222. The samples were preserved at −80°C for use.

### 2.7 Histopathology

Histology of the intestine was performed according to Zeng et al. (27). Intestinal tissues are dehydrated in an alcohol solution before being embedding in paraffin wax. The slices (5 µm) were colored with hematoxylin and eosin and closed with neutral adhesive. The histomorphological structure of four selected sections was observed with an imaging microscope (Nikon YS100, Japan).

### 2.8 Intestinal biochemical parameter measurement

The intestinal biochemical indexes were used for the lipase assay kit (code: A054-1-1), amylase assay kit (code: C106-1-1), pepsin assay kit (code: A080-1-1), superoxide dismutase assay kit (code: A001-1-2), catalase assay kit (code: A007-1-1), malondialdehyde assay kit (code: A003-1-2), total antioxidant capacity assay kit (code: A105-1-2), and glutathione peroxidase assay kit (code: A005-1-2) to detect the lipase (LPS), α-amylase (AMS), pepsin (PEP), superoxide dismutase (SOD), catalase (CAT), malondialdehyde (MDA), total antioxidant capacity (T-AOC), and glutathione peroxidase (GSH-Px). These kits were purchased from the Institute of Biological Engineering (Nanjing, China, http://www.njjcbio.com/).

### 2.9 Quantitative real- time PCR analysis

The quantitative real- time PCR analysis was conducted based on the study of Zhou et al. (28). In brief, RNA was extract ed from the intestinal tract of *H. otakii* at −80°C using the Trizol method (29). The total RNA concentration and quality are determined using a microspectrophotometer (Uyunpop Photoelectric Technology Co. Shangdong, China). The cDNA was synthesized by reverse transcription using the reverse transcription kits (Baisai Biotechnology Co., Shanghai, China), which used total RNA as a template and stored at −20°C. The primers for genes were designed by Primer 5, using *β*-actin as the housekeeping gene (**Table 2**), and the primers of *β*-actin are based on the study of Diao et al. (30). The reaction system is as follows: upstream primer: 0.6 μL, downstream primer: 0.6 μL, 2 × Talent qPCR premix: 10 μL, cDNA: 1 μL, RNase-Free ddH_2_O: 7.8 μL, for a total volume of 20 μL. After 3 min at 95°C, the annealing was carried out at 60°C for 15 s for 40 cycles, denaturing for about 5 s at 95°C, and the temperature was raised upward from 55°C to 95°C. A melting curve analysis was performed. The presence of individual amplicons was confirmed by agarose gel electrophoresis of the end product. The standard curves were created with six various dilutions (in triplicate). The expression analysis results were subjected to the 2^−ΔΔCT^ approach (31).

**Table 2:** The primer sequences used in the present study.

Gene	Primer sequence (5′-3′)
*p65-F*	GACTGCAAACACGGCTACTA
*P65-R*	TGGCCTCATTCACATCCTTC
*VEGF-A-F*	CCTGCCTTTGGATTGGATTTC
*VEGF-A-R*	ACGTCATGTGGACCTCTTTC
*HIF1-α-F*	GCTGGGTGACATAAGAGAGATG
*HIF1-α-R*	TGAAGGCAGCAGAAGTATGG
*IL-6-F*	GTCTGTATCTGGCCGTGATATG
*IL-6-R*	ATGACCGTTACCTGGAGTTTG
*TNF-α-F*	CTTCTACCAGTACGCACATCC
*TNF-α-R*	AACACTCAGACAGCCATACAC
*MCP-1-F*	CCCACTGATGTGCTGAAGAT
*MCP-1-R*	GTTCCCTCCTGCTGGTAAAT
*Cyclin D1-F*	GCCGAGAAGTTGTGCATCTA
*Cyclin D1-R*	AGGTTCCACTTGAGCTTGTT
*MMP9-F*	CCCACTTTGACGATGATGAGT
*MMP9-R*	GTGCAGGTGGTGTAGGATTT
*β-Actin*	CTGGTCTGGATTGGCTGTGA
*β-Actin*	GGAAGGAAGGCTGGAAGAGG

p65, transcription factor p65; VEGF-A, vascular endothelial growth factor A; HIF1-α, hypoxia-inducible factor 1α; IL-6, Interleukin-6; TNF-α, tumor necrosis factor-alpha; MCP-1, monocyte chemoattractant protein 1; Cyclin D1, G1/S-specific cyclin D1; MMP9, metalloproteinase 9.

### 2.10 Statistical analysis

The one-way ANOVA was performed on the experimental data using SPSS 19.0 software (SPSS, Chicago, IL, USA). Data were expressed in mean ± standard error of the mean (SEM). The normality of the distribution and the Chi-square values of the original data were tested using Kolmogorov –Smirnov and Levene’s test. If at least one hypothesis was not confirmed, a mathematical transformation was applied. Multiple comparisons between groups were performed using Duncan’s method. In the figures, ^*^ *p* < 0.05, ^**^ *p* < 0.01, and ^***^ *p* < 0.001 are shown.

## 3 Results

### 3.1 Construction and analysis of the integrated network model

**Figure 1:**
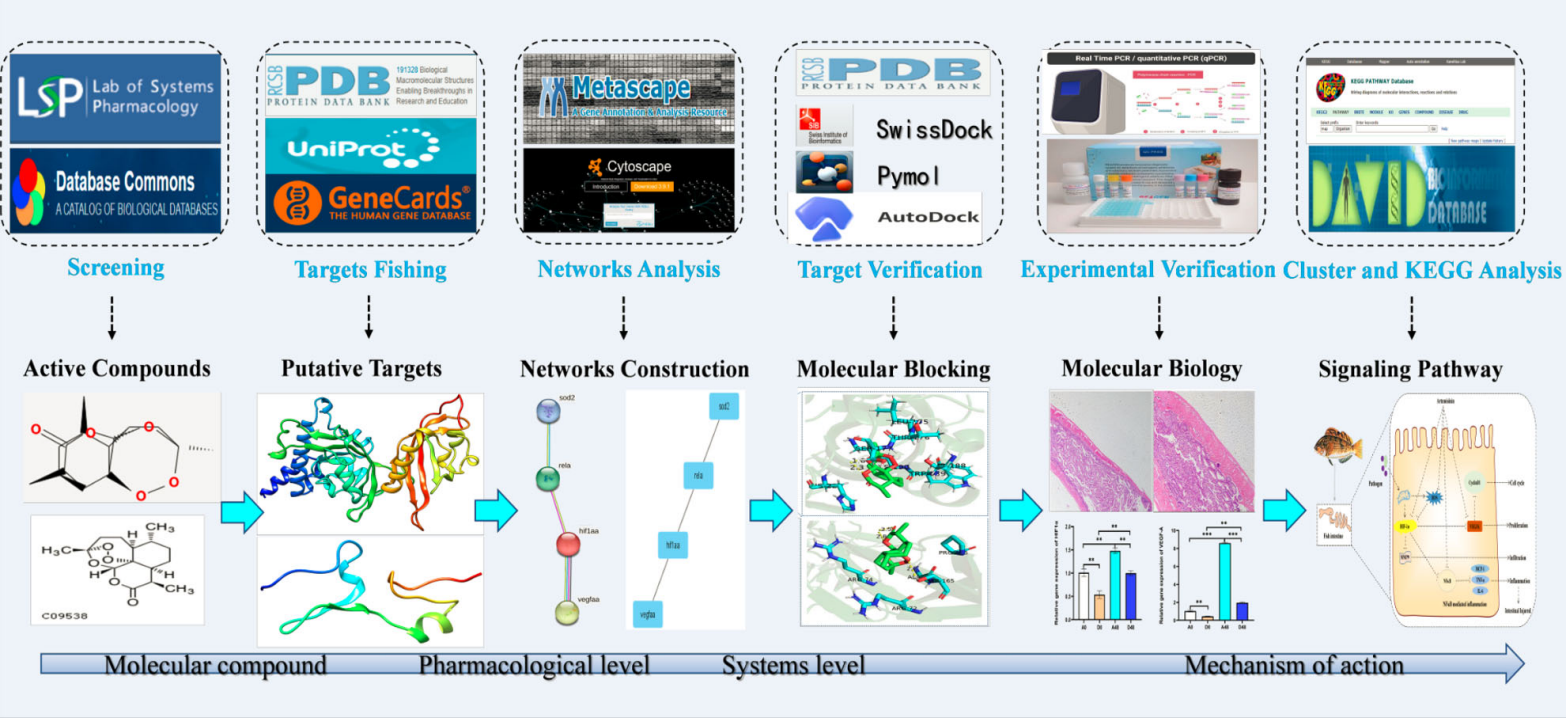
The schematic diagram illustrates the idea and workflow of the study. ART, artemisinin; IID, intestinal immune disease; GO, Gene Ontology; KEGG, Kyoto Encyclopedia of Genes and Genomes; PPI, protein –protein interaction.

**Figure 2:**
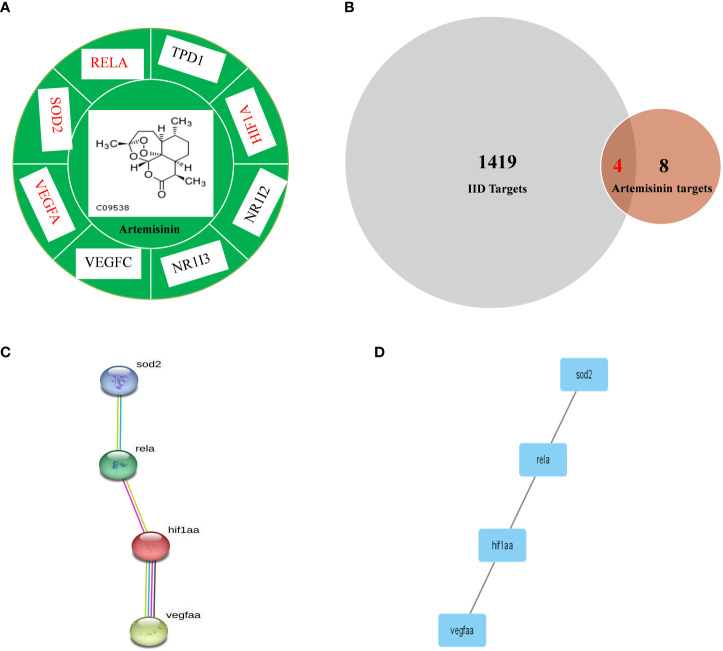
Comparison of IID targets and ART targets. **(A)** ART-target network analysis diagram and types of ART target proteins. **(B)** Venn 2.1.1 diagram of IID-related targets and ART targets; **(C)** The “ART -Target -IID” network diagram. The PPI network of IID targets and ART targets was analyzed by STRING 11.0. Network nodes represent proteins and margins indicate the protein–protein interactions. Known interactions: light blue edges are representative of curation from the database, and pink edges represent an experimental determination; yellow edges are representative of text mining, and black edges are representative co-expression. **(D)** PPI network of protein –protein association was verified by Cytoscape 3.8.2. ART, artemisinin; IID, intestinal immune disease.

**Figure 1** presents the process by which network pharmacology identifies potential mechanisms of action and provides experimental validation of the effects of ART on IID. A number of 15 gene targets were required in the TCMSP database species, and eight gene targets possessed by Zebra fish were filtered in the Uniprot software (**Figure 2A**).

The 209 targets were obtained in the Drugbank database, 44 in the OMIM database, 900 in the Genecards database, and 688 in the PHARMGKB database. After dismissing repeat, 418 goals were then selected. Finally, these goals and eight ART targets were imported into the Venny 2.1.0 platform to obtain four crossover goals (**Figure 2B**).

The four intersecting goals were brought into the STRING 11.0 platform for analysis with a relationship score of > 0.98. The PPI network is illustrated in **Figures 2C, D**. There are four proteins, and one relationship exists between these four proteins. The four pivotal targets are superoxide dismutase [Mn], mitochondrial (SOD2), transcription factor p65 (RELA), hypoxia-inducible factor 1-alpha (HIF1-α), and vascular endothelial growth factor A (VEGF-A).

### 3.2 Exploration of ART molecular mechanism of action

**Figure 3:**
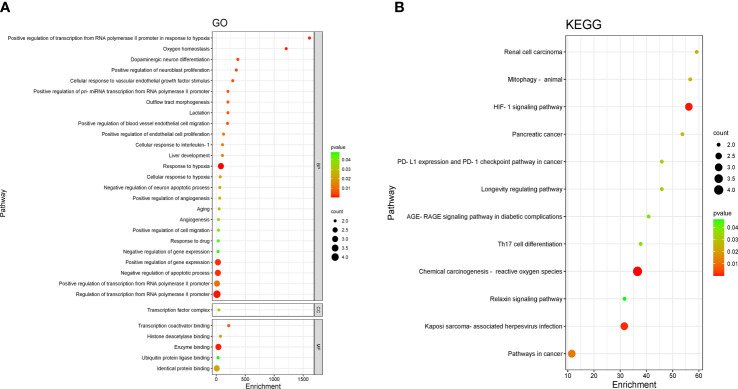
Enrichment analysis of pathways and processes. **(A)** Gene Ontology (GO) functional enrichment analysis. DAVID, the annotation. Visualization and integrated discovery. **(B)** Enrichment analysis of the Kyoto Encyclopedia of Genes and Genomes (KEGG) signaling pathway. The bubble maps of the top 12 bp projects.

The four crossed goals were entered into the DAVID database for GO enrichment analysis. The ranked GO enrichment level entries were picked based on the –log 10 (*p*)-value and a two-dimensional bubble plot was created (**Figure 3A**). The results indicated that there were 37 enrichment results, of which 25 (65.57%) crucial targets were major concentrated in biological processes (BP). GO enrichment analysis identified the first 15 BPs as positive regulation of transcription from RNA polymerase II promoter in response to hypoxia, oxygen homeostasis, dopaminergic neuron differentiation, positive regulation of neuroblast proliferation, cellular response to vascular endothelial growth factor stimulus, positive regulation of pri-miRNA transcription from the RNA polymerase II promoter, outflow tract morphogenesis, lactation, positive regulation of blood vessel endothelial cell migration, positive regulation of endothelial cell proliferation, cellular response to interleukin-1, liver development, response to hypoxia, and cellular response to hypoxia. The one cellular component (CC) term identifies the transcription factor complex. The five molecular function (MF) terms were identified by GO enrichment analysis in the order listed below: transcription coactivator binding, histone deacetylase binding, enzyme binding, ubiquitin protein ligase binding, and identical protein binding.

Four core targets were entered into the DAVID database for enrichment by the KEGG pathway for analysis, and a total of 12 projects were obtained. The two-dimensional bubble chart was drawn using the projects with *p*-value (**Figure 3B**). Among them, the hypoxia-inducible factor-1 (HIF-1) singling pathway and chemical carcinogenesis —reactive oxygen species are the main enrichment areas for the four core targets. Each pathway consisted of different targets in **Table S1**.

### 3.3 Molecular docking

**Figure 4:**
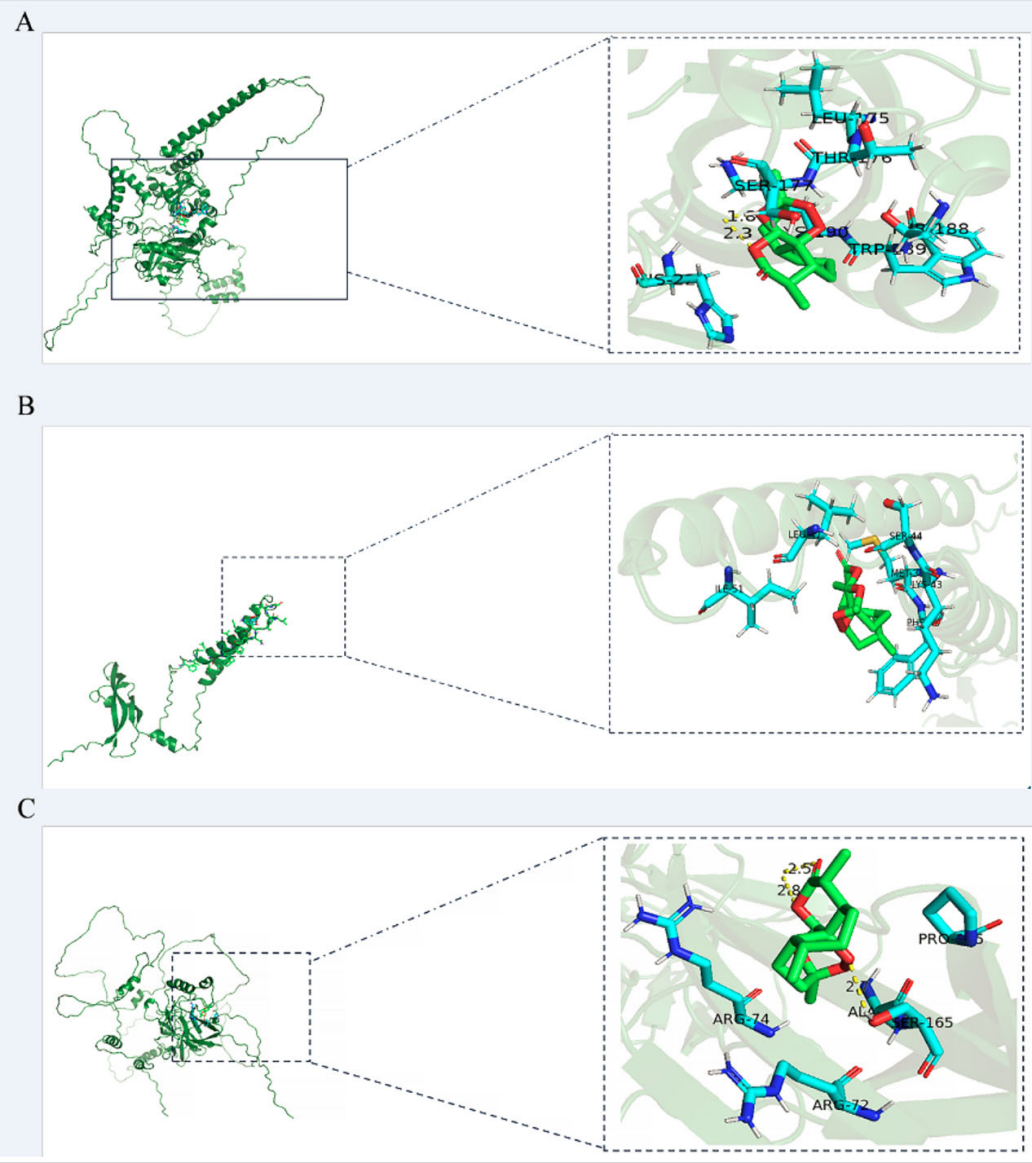
The models of molecular docking, active site, and binding range of molecules are shown with a schematic diagram of ray tracing. **(A)** ART with HIF1-α. **(B)** ART with VEGF-A. **(C)** ART with RELA (p65).

ART was molecularly docked with three targets, including HIF1-α, RELA, and VEGF-A (**Figure 4**; **Table S2**). RELA showed a high degree of binding to ART through three hydrogen bonds and five amino acid residues, resulting in a −6. 7- kcal/mol minimum binding energy. VEGF-A exhibited a high degree of affinity to ART by six amino acid residues involved in hydrophobic interactions, with a minimum binding energy of −6.3 kcal/mol. HIF1-α displayed a higher affinity for ART via two hydrogen bonds and seven hydrophobic residues, with the smallest binding energy of −7.4 kcal/mol.

### 3.4 ART promotes digestion and improves intestinal structure

**Figure 5:**
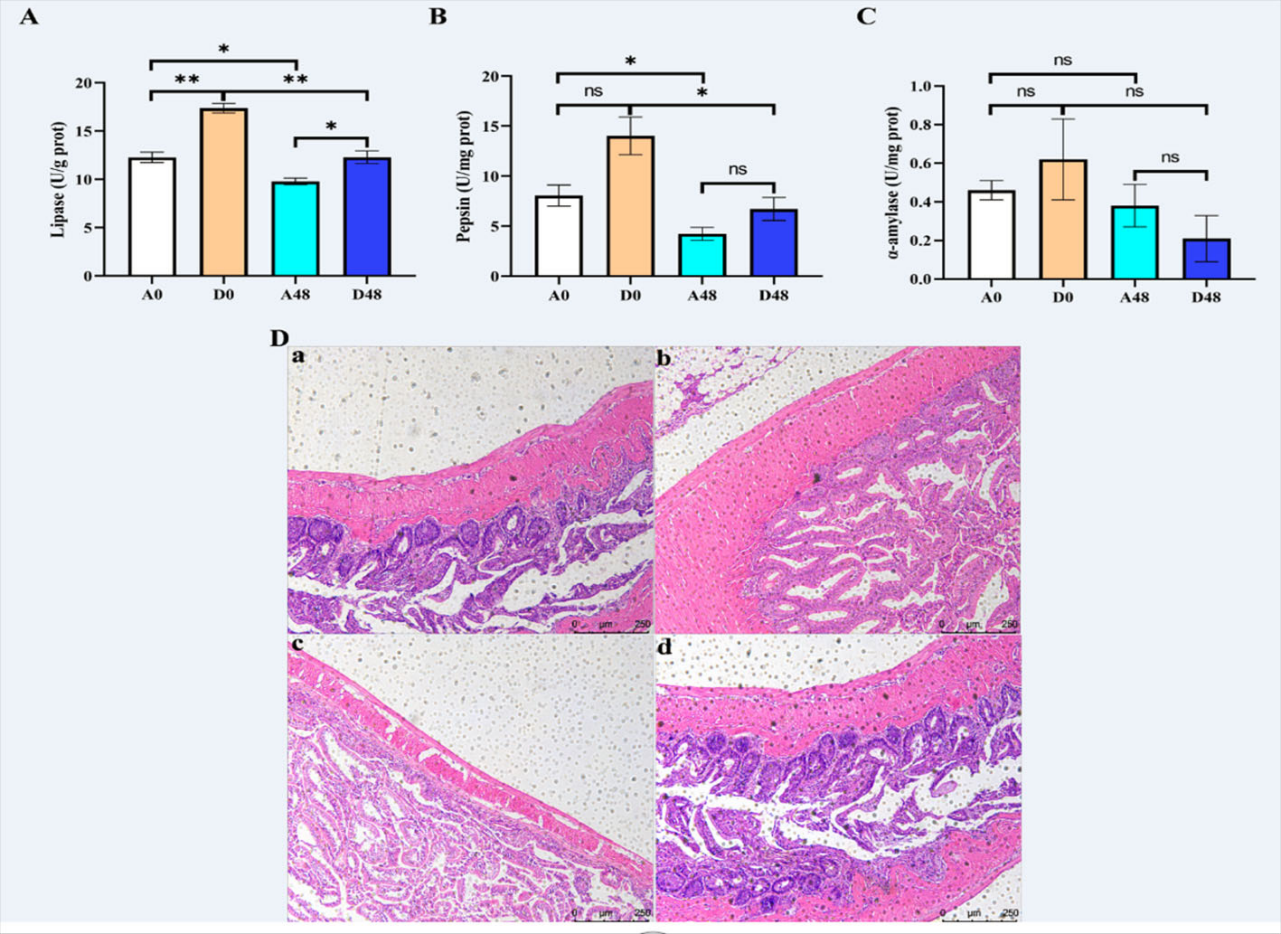
The supplementation of ART with digestive enzymes **(A–C)** alleviated intestinal injury and **(D)** induced by *E tarda* in *H otakii*. Histopathology of the intestines was used to stain the intestines with H&E **(D)**: (a) A0 group; (b) D0 group; (c) A48 group; and (d) D48 group. Mean values for the identical indicator with ^*^ *p* < 0.05 and ^**^ *p* < 0.01 were remarkably different.

The intestinal digestion parameters are presented in **Figures 5A–C**. The activities of LPS and PEP were up-regulated at approximately 1.59- and 1. 32-fold greater in the D48 groups as compared to the A48 groups. There were no significant effects on AMS activities between the groups (*p* > 0.05).

The intestinal mucosa was intact in the A0 group, and the intestinal villi were tightly arranged and largely free of inflammatory cell infiltration. In the A48 group, the intestinal mucosa was broken, the gap between the intestinal villi was increased, and the inflammatory cell infiltration was severe. In the D0 group, the intestinal mucosal structure was restored, the gap between the intestinal villi was reduced, and a few inflammatory cells appeared (**Figure 5**).

### 3.5 ART treatment improved intestinal antioxidant capacity in *H. otakii*

**Figure 6:**
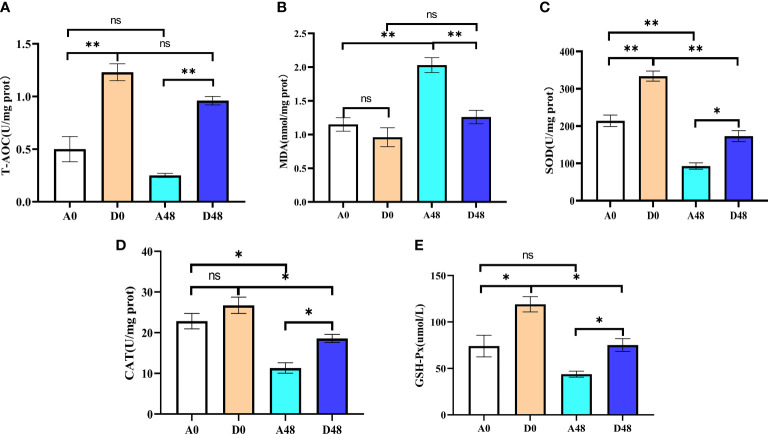
The supplementation of ART on antioxidative indices in the intestinal of *H otakii*. **(A)** The change of T-AOC in the intestinal tract after ART administration and infection of *E tarda.* **(B)** The change of MDA in the intestinal tract after ART administration and infection of *E tarda.* **(C)** The change of SOD in the intestinal tract after ART administration and infection of *E tarda*. **(D)** The change of CAT in the intestinal tract after ART administration and infection of *E tarda*. **(E)** The change of GSH-Px in the intestinal tract after ART administration and infection of *E tarda.* Mean values for the identical indexes with ^*^ *p* < 0.05 and ^**^ *p* < 0.01 were significantly different; ns, no remarkable differences between groups.

The intestinal antioxidant parameters are presented in **Figure 6**. In the A48 group, the SOD and T-AOC were 92.87 and 0.25 U/mg prot, respectively; in the D48 group, their activities were 173.10 and 0.96 U/mg prot. The activities of SOD and T-AOC in A48 were markedly decreased by approximately 1.86- and 3. 84-fold greater as compared to the D48 group (*p* < 0.05). In the A48 group, the activities of CAT and GSH-Px were 11.33 U/mg prot and 43.08 µmol/L; in the D48 group, their activities were 18.57 U/mg prot and 75.15 µmol/L. The activities of CAT and GSH-Px in A48 were markedly downregulated approximately 1.64- and 1. 74-fold greater as compared to the D48 group (*p* < 0.05). Moreover, the content of MDA was 2.03 and 1.26 nmol/mg prot in the A48 group and D48 group, respectively, and the MDA activity was significantly increased by approximately 3. 69-fold greater in the A48 groups compared with the D48 group (*p* < 0.05).

### 3.6 ART modulated the inflammation and activation of the HIF signaling pathways in *H. otakii*

**Figure 7:**
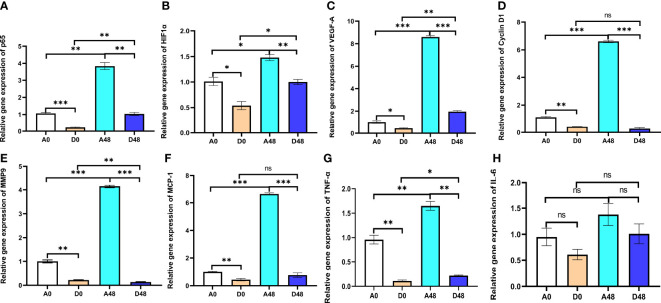
The supplementation of ART with immune genes in the intestinal tract of *H otakii*. **(A)** The gene level of *p65* after *E tarda* infection and ART administration. **(B)** The gene level of *HIF1-α* after *E tarda* infection and ART administration. **(C)** The gene level of *VEGF-A* after *E tarda* infection and ART administration. **(D)** The gene level of *cyclin D1* after *E tarda* infection and ART administration. **(E)** The gene level of *MMP9* after *E tarda* infection and ART administration. **(F)** The gene level of *MCP-1* after *E tarda* infection and ART administration. **(G)** The gene level of *TNF-α* after *E tarda* infection and ART administration. **(H)** The gene level of *IL-6* after *E tarda* infection and ART administration. Mean values for the identical indicator with ^*^ *p* < 0.05, ^**^ *p* < 0.01 and ***p < 0.001. were significantly different ; ns, no remarkable differences between groups.

The intestinal relative gene expression is presented in **Figure 7**. In the A48 group, the mRNA levels of transcription factor p65 (*p65*), *HIF1-α*, and *VEGF-A* were 3.84, 1.48, and 8.60, respectively; in the D48 group, their levels were 1.04, 1, and 1.96. The *p65*, *HIF1-α*, and *VEGF-A* were markedly downregulated, approximately 3.69-, 1.48-, and 4. 39-fold greater in the D48 group as compared to the A48 group (*p* < 0.05). In the A48 group, the *cyclin D1*, monocyte chemotactic protein 1 (*MCP-1*), and matrix metalloprotease 9 (*MMP9*) were 6.61, 6.63, and 4.15, respectively; in the D48 group, their levels were 0.27, 0.77 and 0.14, respectively. The *cyclin D1*, *MCP-1*, and *MMP9* were significantly decreased by approximately 24.48-, 8.61-, and 29. 64-fold greater in the D48 as compared to the A48 group (*p* < 0.05). Moreover, the *TNF-α* was 1.65 in the A48; in the D48 group, its level was 0.22. The *TNF-α* was significantly downregulated approximately 7. 5-fold in the D48 group as compared to the A48 group (*p* < 0.05). Moreover, the expression of *IL-6* in the D48 group showed a decreasing trend compared to the A48 group, although there was no significant difference between the two groups (*p* > 0.05).

## 4 Discussion

To comprehend the mechanism of ART action on IID in *H. otakii*, network pharmacology, molecular docking, and experimental validation were deployed to illuminate the potential action and underlying mechanism of ART in treating IID in *H. otakii*. Network pharmacology provides an avenue to deeply probe into the components and targets of drugs, thereby facilitating their impact analysis (32). In the present study, a total of eight drug targets through searches in the TCMSP database and the UniProt database were procured. ART, the chief active component of *Artemisia annua*, was found to have four common targets with IID. These targets —SOD2, RELA, HIF1-α, and VEGFA —were utilized to construct the PPI network. Furthermore, the GO enrichment analysis of the hub gene in BPs indicated that the cross-targets were chiefly linked to responses to oxidative stress, inflammation, hypoxia, and angiogenesis, which aligns with the pathological process of IID (33–35). Subsequent exploration via the KEGG pathway disclosed that the four core targets were primarily concentrated in the chemical carcinogens, reactive oxygen species, and HIF-1 signaling pathway. Crucially, molecular docking results validated that ART has a pronounced effect on the alleviation of IID, providing an excellent foundation for subsequent fish experiments. This process revealed that ART could bind well to these three target proteins. The HIF1-α and RELA proteins established more stable hydrogen bonds with ART, which allows the ART ligand to bind stably to the active site of the corresponding protein. Simultaneously, hydrophobic interactions exist between the small molecular ligands and protein residues, which serve to augment the stability of compounds in the protein’s active pocket (36).

In fish, the intestine holds substantial responsibility for nutrient digestion and absorption, as well as disease defense. It is accounted for approximately 70% of the immune function of fish (23, 37). Fish IID is strongly correlated with its intestinal digestibility (38). For instance, the digestive enzymes pepsin, lipase, and α-amylase have a vital role in the digestion and absorption of nutrients, which is one of the methods to measure the dietary adaptability of fish (39). PEP is a digestive protease that breaks down protein in the dirt into small peptide fragments (40). LPS is a kind of enzyme with a variety of catalytic capacities that can catalyze hydrolysis, alcoholysis, esterification, transesterification, and the reverse synthesis of triacylglycerol esters and other soluble esters (41). The AMS is found mainly in the digestive system of aquatic animals (42). This study indicated that the activities of LPS and PEP in the D0 and D48 groups were higher than those in the A0 and A48 groups, which suggests that ART could improve the digestive capacity of *H. otakii*. Moreover, the intestine is the body’s first barrier against pathogenic bacteria, and the intestinal villi and mucus immune complexes play a vital role in intestinal function. In fish, the composition of intestinal epithelial cells and colonies is essential for intestinal immunity. The pathogenic bacteria stimulate intestinal immunity by disrupting the intestinal barrier through mucus secreted by cupped cell inhibition as well as by antimicrobial proteins, chemokines, and cytokines (43). Also, pathogenic bacteria can attack *in vivo* immunity by disrupting the composition of intestinal microorganisms through signals from intestinal immune cells, thereby inhibiting the maturation of immune tissues, antibody production, differentiation of T cells, and activation of the phagocytic response of macrophages (44, 45). It was found that the intestinal injury in the D48 group was relieved, inflammatory cells were reduced, and the villus space was tight in the intestine of *H. otakii*, which all suggest that ART could ameliorate the IID of *H. otakii* by improving its intestinal structure.

In this study, the PPI protein interaction network map showed that artemisinin may improve IID through the SOD2/NFκB/HIF1-α/VEGF-A signaling pathway. IID can cause excessive reactive oxygen species (ROS) production, which destroys the intestinal antioxidant system and leads to intestinal oxidative stress (46). ROS plays different roles in cell and tissue health, especially in oxidative stress (47). Fish IID disrupts the balance of ROS, which results in oxidative stress and damage to host DNA, thus inducing protein oxidation and cell damage (48). In fish, ROS are dissolved into water and oxygen molecules by enzymes such as SOD, CAT, and GSH-Px (49). SOD and CAT are significant enzymes that catalyze the transformation of highly active superoxide radicals into hydrogen peroxide or oxygen molecules (50). As a superoxide producer, SOD2 encodes manganese superoxide dismutase in the mitochondrial matrix and secreted superoxide dismutase. SOD2 has a critical position in phagocyte counts and innate immunity in fish by regulating mitochondrial superoxide (51). GSH-Px removes hydrogen peroxide and lipid peroxides from the body (52). MDA comes from the interpretation of reactive oxygen species in polyunsaturated lipids and indirectly reflects the damage of cellular oxidative stress (53). T-AOC reflects the ability of the fish body to inhibit the formation of lipid peroxide (54). This study showed that the activities of SOD, CAT, T-AOC, and GSH-Px were downregulated, and MDA was increased in the intestinal tract in the A48 groups compared with the A0 groups. This situation was changed in the D48 groups, indicating that ART could enhance the antioxidant performance of the intestinal tract of the IID in *H. otakii*.

ART allows for cell surface binding to certain toxins, viruses, and fungi, thereby reducing the antigen’s absorption and boosting the animal’s cellular and humoral immune reactions (55, 56). NFκB is an essential transcription factor that is subject to regulation by intracellular redox status, which can be induced by ROS to the expression of genes involved in immune and cellular resistance networks and is a major regulator of intestinal epithelial inflammation and immune homeostasis (57). p65 is one of the combined forms of NFκB proteins (58), and it has a vital function in the pathogenesis of chronic intestinal inflammation (59). Moreover, IID activates downstream proinflammatory factors such as IL-6 and TNF-α, which are mainly regulated by NFκB-activated endotoxemia by upregulating the levels of IL-6 and TNF-α, inducing intestinal mucosal injury and chronic inflammatory bowel disease (60). On the other hand, the imbalance of the activities of these inflammatory cytokines will destroy the relative stability of the intestinal flora, thereby activating the intestinal epithelial HIF-1 signaling pathway (61), which reflects the intestinal resistance to exogenous bacteria (62). MCP-1 is a small inducible chemokine that is an effective chemotactic agent for monocytes, T lymphocytes, natural killer cells, basophils, and dendritic cells and whose processes can promote cell infiltration and inflammation (63). VEGF-A is one of the target genes of the HIF1-α signal pathway, which is considered to be a major cytokine related to angiogenesis (64). Scaldaferri et al. (65) point out that VEGF-A triggers IID by inducing intestinal angiogenesis and inflammation. VEGF-A, as the target gene protein of HIF-lα, can promote the growth of vascular endothelial cells and enhanced the penetration of vasculature (66). MMP9 is the most complex family of matrix metalloproteinase and has a vital position in cell infiltration (67). MMP9 degrades extracellular matrix components and promotes tissue remodeling, thereby activating the binding of VEGF-A (57). Cyclin D1 has a regulatory effect on the cell cycle. Mutation and overexpression of the *cyclin D1* gene can change the process of the cell cycle and contribute to proliferation. The activation of the VEGF-A-induced mammalian TOR (mTOR) signaling cascade can promote the growth of immune cells by activating cyclin D1 (68). In this study, supplementation with ART at 600 mg/kg resulted in decreased mRNA levels of *p65*, *HIF1-α*, *VEGF-A*, *IL-6*, *TNF-α*, *MCP-1*, *MMP9*, and *cyclin D1* in the intestinal tract of *H. otakii* after *E. tarda* infection. These results suggest that ART could improve IID in *H. otakii*.

**Figure 8:**
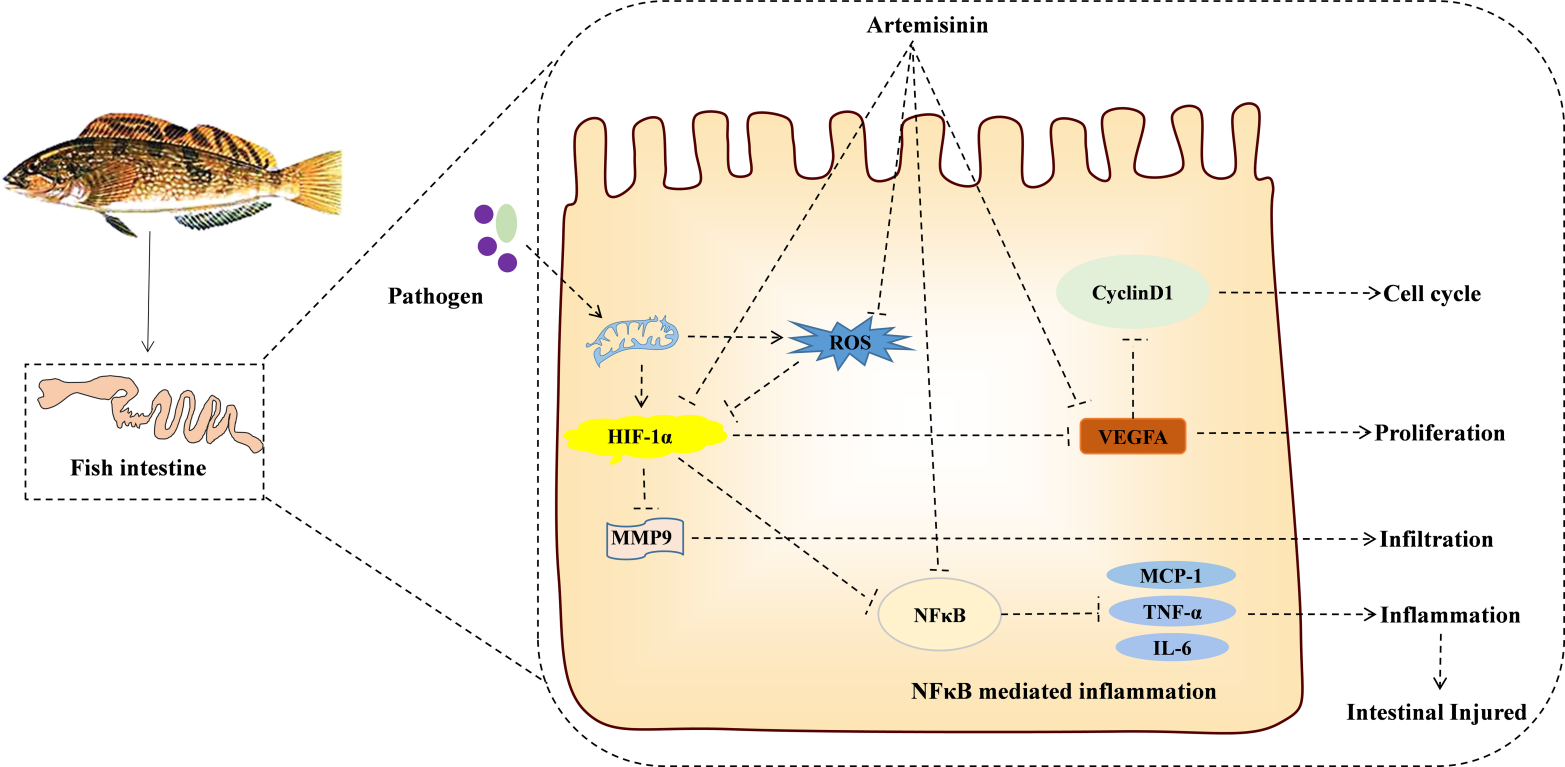
KEGG enrichment pathway revealed the interaction between ART and IID of *H. otakii*. It is possible that ART inhibits the SOD2/NFκB/HIF1-α/VEGF-A pathway by enhancing antioxidant capacity and then inhibiting cell proliferation (cyclin D1), infiltration (MMP9), angiogenesis (VEGF-A), and the levels of major proinflammatory cytokines (TNF-a, IL-6, and MCP-1), which are important in preventing the acute intestinal damage caused by *E. tarda* and maintaining intestinal function.

This investigation uncovers the potential mechanism of action for ART in addressing IID, utilizing a network pharmacological approach. This illuminates a further theoretical foundation (SOD2/NFκB/HIF1-α/VEGF-A) for appraising the protective mechanism exerted by ART on the intestine of *H. otakii* (refer to **Figure 8**). When supplemented at a dosage of 600 mg/kg in diets, ART significantly enhanced the immune capabilities, along with fostering improved digestion and growth in *H. otakii* (data not presented). In essence, dietary ART can be deployed as an efficacious additive to augment intestinal health in *H. otakii* and alleviate intestinal immune disease.

## Data availability statement

The original contributions presented in the study are included in the article/**Supplementary Material**. Further inquiries can be directed to the corresponding author/s.

## Ethics statement

The animal study was reviewed and approved by The Animal Ethics Committee of Dalian Ocean University (Dalian, China) approved all experimental protocols used in this study. All animal procedures follow the “Guidelines for Ethical Treatment of Experimental Animals” prepared by the Ministry of Science and Technology of China. The anatomical experiments were conducted under anesthesia with 3-aminobenzoate ethyl methanesulfonic acid (MS-222, Sigma-Aldrich, USA), minimizing the pain of fish.

## Author contributions

YG, XW, YS, DC, and YZ designed the experiments and supervised the manuscript. TP, WJW, JH, XL, and YC carried out the animal experiment and sample analysis with the help of ZX. WW wrote the manuscript. All authors read and approved the final manuscript. All authors contributed to the article and approved the submitted version.
