# Supplementary material for: Dietary artemisinin boosts intestinal immunity and healthy in fat greenling (Hexagrammos otakii)
Source: Front Immunol. 2023 Jul 17;14:1198902. doi: 10.3389/fimmu.2023.1198902 (PMC10388541; doi:10.3389/fimmu.2023.1198902)
Supplement: Supplementary file 1 [file DataSheet_1.docx]

**
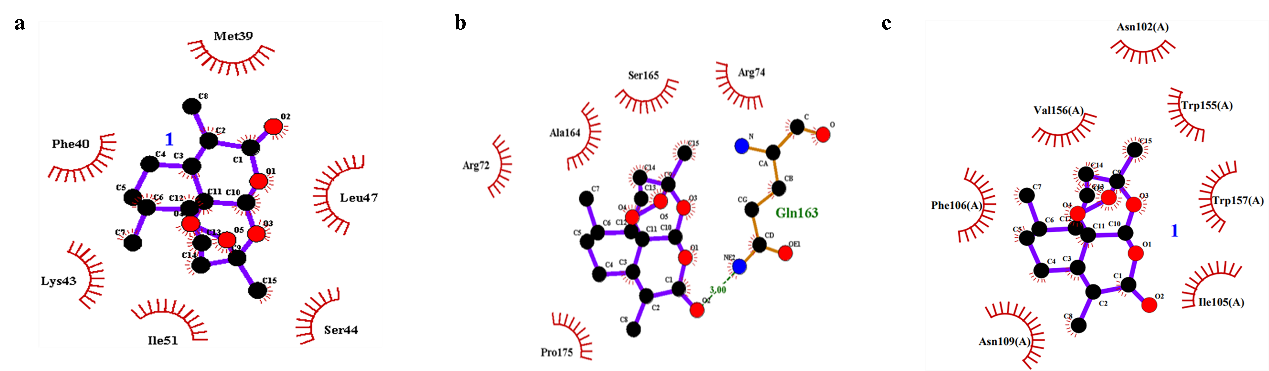
**

**Figure 1.** Schematic 2D representation that the molecular docking model, active sites and binding distances, (A). ART in the protein HIF-1α, (B). ART with VEGF-A, (C). ART with RELA (p65), respectively.
