# Supplementary material for: Dietary artemisinin boosts intestinal immunity and healthy in fat greenling (Hexagrammos otakii)
Source: Front Immunol. 2023 Jul 17;14:1198902. doi: 10.3389/fimmu.2023.1198902 (PMC10388541; doi:10.3389/fimmu.2023.1198902)
Supplement: Supplementary file 2 [file DataSheet_2.docx]

The amino acid sequence of the Fat greenling used for molecular docking is as follows:

>HIF-1

MDSGTAPEKKRVSSERRKEKSRDAARSRRGKESEVFYELAQELPLPHSVSSSLDKASIMRLIISYLRMRKVLSTDEPMTEEETELESQLNSSYLKALEGFLLVLSEDGDMIYLSENVNKCLGLAQFDLTGHSVFDYIHPCDQEELREMLVHKTGSKKAKEPNTERSFFLRMKCTLTSRGRTVNVKSATWKVLHCSGHVRVYDTHTEETPNGHSEPAVPYLVLICDPIQHPSNIEVPLDTKTFLSRHTMDMKFTYCDERITELLGYDPEDLLNRSVYEYYHALDSDHLTKTHHNLFAKGQVSTGQYRMLAKRGGFVWVETQATVIYNNKNSQPQCVVCVNFVLSGIQEEKLILSLEQIEDVKPVKEEEEEQQEEEEKEVVESIQPDTSPALLKDEKKGPERDVIKLFTQAVEAQPLASLYDQLKAEPEALTLLAPAAGDAIISLDFSCPDSEIQLPKEVPLYNDVMLPSTSDKLALPLSPLPLSEPLRVSSTSSEEAEAKSYAPAGSTSSTNRSSSQADSPLDFCYPMDSEMSSDFKLDLVEKLFAIDTEPKTPFTTQAMEDLDLEMLAPYIPMDDDFQLRSLTPEESLSCGPVKSLQSSPVHVPQDIQSYPSSPFSSPGSRTTSPAPLAAPRPATIVATRTPQLDKDVSLRTLVAQISQRKRKLGDIREMMGQEVVPQEQVEPGKKLKASETTRTILLLPSDLASRLLGSTSEGTASLCTLPQLTSYDCEVNAPLQGRQSLLQGEELLCALDHINSI*

>VEGF-A

MLRPRVRHGFAITTPCPLFREVLAGPLVLSFLLFFLNFMFENKSPLLASWILKRHLPLDWISNTMNIIDSLTLLFLTLSAVKSAHIPKEPERGPHDVIPLMEVYNKSLCQPRELLVEILQEYPEEVEHIFIPSCVVLTRCAGCCNDEMLQCMPTSTYNITMEIKRIKPQRQQNDIFMSFTEHSACECRLKKEVKEPKEK*

>p65

MDGVYGWGLTTLNPVQAASPFIEIIEQPKQRGMRFRYKCEGRSAGSIPGEKSNDTTKTHPAIKMHNYSGPLRVRISLVTKNAPHKPHPHELVGKDCKHGYYEADLQERRVHSFQNLGIQCVKKKDVNEAITCRLQTNNNPFNIPEAKVWEEEFDLNSVRLCFQASITLASGDLIPLEPVVSQPIYDNRAPNTAELKICRVNRNSGSCKGGDEIFLLCDKVQKEDIEVRFFQDSWEGKGTFSQADVHRQVAIVFRTPPYRDTNLSEPIRVKMQLRRPSDREVSEPMDFQYLPADPDEYRLSEKRKRTGDMFQSLKLGPMLSSVSMPQDRRHISPARRTVTAKPPSMNAQVAVVAPPGASGAKAQPSYSYQPGQLFSVQPKVEAISAATTNQTWRIMESLNLGPQPKATPVANFTMSQATALCSTTSTSTANQDYSTVNMSDLHQFFPNISSAMAQETAASQGSSASSQTGISFTLPGSQFHVDAPLADDDIPEFPSFSEAQAQGTLENLNMDDFEDLLNPVLMNVSGNGSSMLAQASCQQAAPLGSSTASHSAASQNTSDPASIPGSTWMNYPNSIVNLLQNEGMIDNGNHRPPVLDEFDELMSADEDRLISIFNSGSQAGFVSGHPT*
