# Supplementary material for: Dietary artemisinin boosts intestinal immunity and healthy in fat greenling (Hexagrammos otakii)
Source: Front Immunol. 2023 Jul 17;14:1198902. doi: 10.3389/fimmu.2023.1198902 (PMC10388541; doi:10.3389/fimmu.2023.1198902)
Supplement: Supplementary file 3 [file Table_1.docx]

**Table S1.** KEGG analysis of the 12 pathways.

| **ID** | **Pathway** | **Gene** |
| --- | --- | --- |
| hsa05211 | Renal cell carcinoma | HIF1A, VEGF-A |
| hsa04137 | Mitophagy - animal | HIF1A, RELA |
| hsa04066 | HIF-1 signaling pathway | HIF1A, RELA, VEGF-A |
| hsa05212 | Pancreatic cancer | RELA, VEGF-A |
| hsa05235 | PD - L1 expression and PD - 1 checkpoint pathway in cancer | HIF1A, RELA |
| hsa04211 | Longevity regulating pathway | SOD2, RELA |
| hsa04933 | AGE - RAGE signaling pathway in diabetic complications | RELA, VEGF-A |
| hsa04659 | Th17 cell differentiation | HIF1A, RELA |
| hsa05208 | Chemical carcinogenesis - reactive oxygen species | SOD2, HIF1A, VEGF-A, RELA |
| hsa04926 | Relaxin signaling pathway | RELA, VEGF-A |
| hsa05167 | Kaposi sarcoma - associated herpesvirus infection | HIF1A, RELA, VEGF-A |
| hsa05200 | Pathway in cancer | HIF1A, RELA, VEGF-A |
