# Supplementary material for: Dietary artemisinin boosts intestinal immunity and healthy in fat greenling (Hexagrammos otakii)
Source: Front Immunol. 2023 Jul 17;14:1198902. doi: 10.3389/fimmu.2023.1198902 (PMC10388541; doi:10.3389/fimmu.2023.1198902)
Supplement: Supplementary file 4 [file Table_2.docx]

**Table S2.** The results of molecular docking.

| **Protein** | **Binding energy (KJ/mol)** | **hydrogen** | **Amino acid residues** |
| --- | --- | --- | --- |
| HIF1A | -7.4 | 2 | 7 |
| RELA | -6.7 | 3 | 5 |
| VEGF-A | -6.3 | - | 6 |
