# Supplementary material for: Dietary artemisinin boosts intestinal immunity and healthy in fat greenling (Hexagrammos otakii)
Source: Front Immunol. 2023 Jul 17;14:1198902. doi: 10.3389/fimmu.2023.1198902 (PMC10388541; doi:10.3389/fimmu.2023.1198902)
Supplement: Supplementary file 5 [file Table_3.docx]

| **Protein name** | **Accession Numbers** | **Links** |
| --- | --- | --- |
| P65 | B3DHW2 | https://www.uniprot.org/uniprotkb/B3DHW2/entry |
| HIF1A | Q6EHI4 | https://www.uniprot.org/uniprotkb/Q6EHI4/entry |
| VEGFA | O73682 | https://www.uniprot.org/uniprotkb/O73682/entry |
| NR1I2 | BOV1H8 | https://www.uniprot.org/uniprotkb/B0V1H8/entry |
| TPD1 | F8W5U2 | https://www.uniprot.org/taxonomy/7955 |
| VEGFC | Q7T316 | https://www.uniprot.org/uniprotkb/Q7T316/entry |
| SOD2 | Q6P980 | https://www.uniprot.org/uniprotkb/Q6P980/entry |
| NR1I3 | [A0A8M9QAV5](https://www.uniprot.org/uniprotkb/A0A8M9QAV5/entry) | https://www.uniprot.org/uniprotkb/A0A8M9QAV5/entry |

Table S3 The information of target genes
